# Bortezomib a Safe Treatment for Patients with Multiple Myeloma and Cystic Fibrosis

**DOI:** 10.4084/MJHID.2012.035

**Published:** 2012-05-07

**Authors:** Fabiana Gentilini, Anna Levi, Vincenzo Federico, Eleonora Russo, Robin Foà, Maria Teresa Petrucci

**Affiliations:** Hematology, “Sapienza” University of Rome. Via Benevento 6, 00161 Rome (Italy)

## Abstract

**Introduction:**

Bortezomib is a proteasome inhibitor that targets myeloma cell and its bone marrow microenvironment. Intravenous Bortezomib with or without dexamethasone, is effective and well tolerated in patients with relapsed/refractory multiple myeloma (MM).

**Methods:**

We used Bortezomib without corticosteroids, to avoid the risk of lung infection reactivations due to patient’s *Pseudomonas aeruginosa* colonization, in a MM patient with Cystic Fibrosis. Four 21-day cycles of Bortezomib were administrated at 1.3 mg/m^2^ on days 1,4,8 and 11 with a 10 day rest period. Treatment response and toxicity were evaluated.

**Results:**

After four cycles of therapy the patient achieved a very good partial response (VGPR) according to the IMWG response criteria, without clinically significant side effects.

**Conclusions:**

Bortezomib can be successfully utilized for the management of this difficult disease situation.

Bortezomib is a proteasome inhibitor that targets myeloma cell and its bone marrow micro-environment, inhibiting the binding and the paracrine loops between myeloma and bone stromal cells, as well as demonstrating anabolic effects on the bone.

Intravenous Bortezomib (1.3 mg/m^2^ administered on days 1,4,8 and 11 of a 21 day cycle), with or without dexamethasone, is effective and well tolerated in patients with relapsed/refractory multiple myeloma (MM), as demonstrated by the phase II CREST^1^ and SUMMIT^2^ trials, and the phase III APEX^3^ trial. Bortezomib is also effective and well tolerated as part of a first line regimen in untreated myeloma patients as established by the Italian VISTA^4^ and GIMEMA^5^ trials. The most frequently reported extra-hematological adverse events associated with the use of Bortezomib are peripheral neuropathy, gastrointestinal events, fatigue, and Varicella-Zoster virus (VZV) reactivation. Acyclovir prophylaxis at 400–600 mg per day may prevent early VZV reactivation.

We hereby describe a case of MM diagnosed in a patient with Cystic Fibrosis (CF); our patient was treated with Bortezomib therapy without any important side effect.

A 36-year-old man was admitted to our Centre in October 2009 with anemia (Hb 10,4 g/dl, MCV 78 fl, RBC 3.660.000/mm^3^, WBC 8.300/ mm^3^, platelets 273.000/mm^3^) and a monoclonal protein (1,78 g/dl). The patient received a diagnosis of CF in adult age. Since childhood he reported a history of persistent lung infections and bronchiectasis. In 2003 a diagnosis of CF was made by studying the mutations of the gene encoding the CFTR protein, which functions as a chloride channel within a number of epithelial tissues, typically involved in this disease. He showed a CFTR exocrine pancreatic insufficiency and fat malabsorption, as in 90% of adult CF patient,^6^ and a CF-related diabetes (40% of adults)^7^ treated with insulin. As CF-related bone disease he presented osteoporosis, common in adult CF patients,^8^ treated with calcium and biphosphonates. A CT scan performed in August 2009 showed a fracture of D6 that was initially referred to the low bone mineral density. In September 2009, the patient was addressed to our Centre due to a presence of anemia and of a monoclonal component. We confirmed the anemia (Hb 10.4 g/dl) and the paraproteinemia (1.9 g/dl) with total serum protein levels of 8.4 g/dl and albumin 4.6 g/dl. Immunofixation study detected a IgA-k band in the serum and urinalysis was positive for the Bence Jones protein with 10.3 g/24h k light chains. β_2_ microglobulin was elevated at 4547.3 mcg/L (normal values 700–3400) and the lactate dehydrogenase level was normal 112 U/L (135–225). The other laboratory results including complete blood count, electrolytes, liver and kidney function tests, were normal. A peripheral blood smear showed rouleau formation of red blood cells, but no morphologically abnormal cell. Bone marrow aspiration and biopsy were performed and revealed a moderate dysplastic plasmacytosis of 60% and a mild hypercellular marrow with a patchy infiltration of mature and immature CD138+ plasma cells. The immunohistochemical stain for λ and κ showed clusters of dysplastic plasma cells with monotypic k light chain marking. There was no evidence of lytic lesions on X-ray films, but a CT-scan performed in August 2009 showed multiple lytic lesions at the dorsal vertebra and pathologic fractures. A diagnosis of MM was made and the patient was classified as stage IIIA and II according to Durie and Salmon and ISS, respectively. We decided to treat the patient with Bortezomib alone to avoid the use of corticosteroid and the risk of lung infection reactivations, due to his *Pseudomonas aeruginosa* colonization. Four 21-day cycles of Bortezomib were administrated at 1.3 mg/m^2^ on days 1,4,8 and 11 with a 10 day rest period. As antibacterial and antiviral prophylaxis the patient received Ciprofloxacine 1000 mg and Acyclovir 600 mg daily. He had no significant adverse events during the treatment. A re-evaluation carried out in February 2010, after four cycles of therapy, showed a disappearance of the M protein in the serum and urine, the presence of an abnormal IgA-k band in the serum immunofixation analysis, and a concomitant decrease of bone marrow plasma cells to 7%. This met the definition of a very good partial response (VGPR) according to the IMWG response criteria.^9^ Based on these results, the patient was treated with subcutaneous G-CSF (600 mcg per day) to mobilize his hematopoietic stem cells that were successfully collected from his peripheral blood by leukoapheresis with a yield of 10 × 10^6^ CD34+ cells per kilogram of body weight. Based on the excellent response and on his past medical history, we decided to delay the autologous stem cell transplantation eventually at the time of relapse. To date, the patient is maintaining his response after a follow-up of 13 months.

MM is a B-cell malignancy characterized by the expansion of malignant plasma cells in the bone marrow and, in some case, by the concomitant presence of an extramedullary involvement. Only 2% of MM cases are identified in patients younger than 40 years. To the best of our knowledge, there are no reported cases of a MM diagnosed in patients with CF.

However, a mismanagement of protein folding and function during membrane trafficking through the exocytic and endocytic cell pathways by the proteostasis network is responsible for a wide range of diseases that include lysosomal storage diseases, myelination diseases, CF, systemic amyloidosis and light chain myeloma.^10^

Bortezomib is the first clinically approved proteasome inhibitor for patient with relapsed and/or refractory MM. His effectiveness was established in the phase III APEX^3^ trial in terms of both response and time to progression compared to high dose dexamethasone. Data from phase II/III trials in untreated MM patients confirmed the efficacy and safety of Bortezomib alone or in combination with other antimyeloma drugs.^11^,^12^ Based on the consideration that patients with CF can present acute respiratory complications that are the most challenging problems and account for more than 90% of deaths in these patients,^13^ we decided to use Bortezomib alone, in this young patient with CF who received a diagnosis of MM, in view of the heavily immunocompromized status of the patient and his history of frequent lung infection reactivations and Pseudomonas colonization. We obtained a marked and persistent response without important side effects, suggesting that Bortezomib can be successfully utilized for the management of this difficult disease situation.
